# Artificial Arms and Legs

**Published:** 1865-12

**Authors:** 


Artificial Arms and Legs.—The soldiers who find themselves in want of legs or arms will be interested in the advertisement in the present Journal of Dr. Hudson, who furnishes these very necessary instruments gratuitously.

Wm. H. Peabody, druggist, in this city, is agent, and physicians having such soldiers under their care are invited to call their attention to the fact that Government will give them a leg or arm; they can call upon Mr. Peabody for particulars.

